# Relationship of plasma MBP and 8-oxo-dG with brain damage in preterm

**DOI:** 10.1515/med-2022-0566

**Published:** 2022-10-21

**Authors:** Yuwei Zhao, Guanghui Liu, Lei Liang, Zaiwei Yu, Jian Zhang, Hong Zheng, Liying Dai

**Affiliations:** Neonatology Department, Anhui Provincial Children Hospital, Hefei, China; Pulmonary Department, Anhui Provincial Children Hospital, Hefei, China; Neonatology Department, Fuyang First People’s Hospital, Fuyang, China

**Keywords:** myelin basic protein, 8-oxo-deoxyguanosine, preterm infants, white matter injury, periventricular–intraventricular hemorrhages, brain injury

## Abstract

Preterm infants face a significant risk of brain injury in the perinatal period, as well as potential long-term neurodevelopmental disabilities. However, preterm children with brain injury lack specific clinical manifestations in the early days. Therefore, timely and accurate diagnosis of brain injury is of vital importance. This study was to explore the diagnostic efficiency of myelin basic protein (MBP) and 8-oxo-deoxyguanosine (8-oxo-dG) serum levels in brain injury of premature infants. A total of 75 preterm infants with gestational age between 28 and 32 weeks and birth weight higher than 1,000 g were prospectively included. MBP serum levels were significantly higher in premature infants with white matter injury (WMI). 8-oxo-dG serum levels were significantly increased in both WMI and periventricular–intraventricular hemorrhages (PIVH). MBP and 8-oxo-dG were significantly correlated. The area under the curve was 0.811 [95% confidence interval (CI) 0.667–0.955; *p* = 0.002] in MBP and 0.729 (95% CI 0.562–0.897; *p* = 0.020) in 8-oxo-dG. Therefore, the results showed that high MBP levels indicated a possibility of WMI in the premature brain during the early postnatal period, while high 8-oxo-dG levels were closely related to both WMI and PIVH, thus suggesting that MBP and 8-oxo-dG could be used as potential neuro-markers of preterm brain injury.

## 1 Introduction

Following the rapid development of obstetric and neonatal intensive care technology, preterm birth and survival rates have markedly increased; yet, this did not lead to a decrease in severe acute and chronic morbidity and disability in preterm infants [1,2], which include respiratory outcomes, feeding problems, and neurological sequelae [3,4,5]. WHO reported that preterm-related complications were the fourth leading cause of “loss of healthy years of life” due to disability worldwide. The most harmful neurological outcomes related to prematurity include cerebral palsy, learning impairment, and visual disorders, all of which have a negative long-term effect on physical health. The common type of brain injury at early postnatal age is divided into hemorrhagic and non-hemorrhagic injury. In preterm infants, brain injury mainly manifests as periventricular–intraventricular hemorrhage (PIVH) and white matter injury (WMI), which are the leading causes of the nervous system and mental development disorders in very and extremely preterm infants in developed countries [6]. Brain injury is commonly detected with cranial ultrasound (cUS) and magnetic resonance imaging (MRI). However, these approaches have some limitations, such as cUS insensitivity to WMI and MRI being inconvenient for use in preterm infants [7]. Since it can be very challenging to establish a certain final diagnosis in the early period, such as premature encephalopathy, it is very important to identify relatively simple and effective biological indicators for early detection of preterm brain injury that would allow for timely imaging examination and treatment, thus avoiding the deterioration of brain injury.

The immature brain undergoes rapid and critical developmental events, including the neuro-process of myelination at 24–32 weeks of gestational age. Myelin basic protein (MBP) is a major protein that is believed to be of great importance in the myelination of oligodendrocyte in the nervous system. Mature oligodendrocytes can synthesize MBP to complete the myelination of nerve fibers. When the myelination process is disturbed by pathological consults, such as hypoxia and inflammation, MBP levels are very low in the body, and MBP can be released into the cerebrospinal fluid (CSF) and blood. The extent of oligodendrocyte glial cell myelination is associated with the MBP levels in blood or CSF [8]. Animal models and clinical studies have proved that MBP is an ideal biochemical neuro-marker for brain injury, especially for myelin damage [9,10].

Besides, oxidative stress had been recognized as a critical risk factor for brain damage in preterm infants [11]. Oxidative stress is defined as the imbalance between prooxidants and antioxidants in the organism. Reactive oxygen intermediates can damage various biomolecular species, such as DNA, lipids, and proteins. 8-oxo-dG has been extensively used as a specific and sensitive biomarker for DNA damage induced by oxidative stress [12,13]. The higher the 8-oxo-dG levels, the more severity in brain injury [14].

The two important triggers of brain injury in preterm infants are obstruction of myelin synthesis and oxidative stress [15]. Many animal models and a few clinical studies have shown that both MBP and 8-oxo-dG have essential implications in the process of premature brain development [8,16,17]. Yet, identifying which of these biomarkers are more sensitive to change after brain injury in preterm infants might reflect the severity of brain damage, as well as the relationship between biomarkers and the common brain injury in preterm infants. Consequently, this study investigated the clinical value of MBP and 8-oxo-dG in the early postnatal period for different types of brain injuries in very preterm. We also clarified the cut-off points of the two biomarkers to ensure the specificity and sensitivity of early diagnosis in brain injury.

## 2 Methods

### 2.1 Inclusion and exclusion criteria

We performed a prospective cohort study that included all premature infants at 28 and 32 weeks of gestational age with a birth weight (BW) higher than 1,000 g within 7 postnatal days who were admitted to our level 3 neonatal intensive care unit in Anhui Provincial Children Hospital between January and August 2016. The included infants were chosen for two reasons. First, the perinatal stage in China is defined as the period between 28 weeks of gestational age (BW higher than 1,000 g) and 7 postnatal days. Very preterm infants with 28 and 32 weeks of gestational age are more fragile to brain injury. Second, the families with the preterm infants with lower than 28 weeks of gestational age face huge mental stress and economic burden; therefore, the incidence of giving up treatment is high. Therefore, this study included the subpopulation at 28 and 32 weeks of gestational age with a BW higher than 1,000 g with most cases and higher incidence of brain injury.

We excluded cases without cUS or MRI results; neonates with chromosomal abnormalities or those who died dropped out of treatment or were transferred. The infants whose parents refused to save blood samples for the determination of MBP and 8-oxo-dG were also excluded.

All parents gave informed written consent for their child to participate in the study. The important notes in the consent are to get authority of blood sample and guarantee the only purpose for the examination of MBP and 8-oxo-dG and the secrecy of the result except for publication. The study was approved by the institutional committee responsible for human experimentation in Anhui Provincial Children Hospital (Nr: EYLL-2017-023).

### 2.2 Markers’ measurements

Blood samples were collected from premature infants in the first postnatal week, which were then tested using the enzyme-linked immunosorbent assay (ELISA). The ELISA reagent kit for MBP was purchased from R&D (USA), and the other ELISA reagent kit for 8-oxo-dG was purchased from Shanghai Westang Bio-tech Co. Ltd (China).

### 2.3 Imaging examination

For included cases, cUS was routinely performed in the first postnatal week and then repeated during 2–3 weeks. Moreover, cranial MRI was performed before discharge or at the correct gestational age of 40 weeks.

cUS diagnostic criteria are as follows: according to the Papile indexing, PIVH was divided into four degrees. Grades III and IV are termed as “severe IVH.” WMI showed localized or widespread hyperechoic reflection in double periventricular at an early stage: the local small cysts changes or extensive cysts changes in periventricular after a few weeks after birth.

Cranial MRI diagnostic criteria are as follows: PIVH showed different signal abnormalities along with time after bleeding in subependymal germinal matrix and (or) intraventricular. WMI showed white matter signal abnormalities or diffuse high T2 signal or low T1 signal.

### 2.4 Group division

Due to the individual limitations in imaging technology, such as cUS insensitivity to WMI and MRI being inconvenient for use in preterm infants, these two examination methods could be combined to provide a more accurate diagnosis of brain injury. All cases were grouped according to the both results of cUS and MRI. No brain injury group was defined as cases with normal cUS and MRI. Brain injury group was defined as cases with PIVH or WMI in cUS or MRI.

### 2.5 Data collection

Perinatal data and clinical complications were extracted from the hospital files by two experienced neonatologists. The included data related to parents were mother’s age, type of delivery (vaginal or cesarean section). The data related to infants included sex, gestational age by weeks calculated from the last menstrual period and confirmed by physical examination (GA), BW, premature rupture of membranes, and abnormalities of fetal attachment structure (placenta, umbilical cord, and amniotic fluid). The data related to clinical complications were asphyxia, presence of respiratory distress syndrome, use of mechanical ventilation, congenital heart disease confirmed by echocardiography, necrotizing enterocolitis, infection, hypoglycemia, retinopathy of prematurity, PIVH (mild: grades I and II, severe: grades III and IV), and WMI.

### 2.6 Statistical analysis

All subsequent analyses were performed with SPSS V14. The 8-oxo-dG values showed a marked asymmetry; thus, natural log transformation was applied. Continuous data with normal distribution were presented as mean ± standard deviation (*x* ± *s*), otherwise as a range. The *t*-test was used for comparing two groups, and AVNOA was used for comparing more than two groups. Categorical data were presented as the number or ratio of cases. Differences in categorical data were analyzed using the *χ* ^2^-test or Fisher’s exact test as needed. Pearson correlation analysis of MBP and 8-oxo-dG was performed. The predictive value of MBP and 8-oxo-dG was determined using the receiver–operator characteristic (ROC) curve. All tests were two sided. A *p*-value of <0.05 was considered statistically significant.


**Ethical approval and consent to participate:** All parents gave informed written consent for their child to participate in the study. The study was approved by the institutional committee responsible for human experimentation in Anhui Provincial Children Hospital (Nr: EYLL-2017-023).

## 3 Results

### 3.1 General information on included patients

A total of 75 preterm infants at 28–32 weeks of gestational age and birthweight >1,000 g were included in the analyses. In the brain injury group, there were 58 cases with brain injury, including 10 cases of WMI and 56 cases of PIVH. The median gestational weeks and BW were 30.38 ± 1.15 weeks and 1499.69 ± 220.45 g, respectively. The media MBP level was 74.63 pg/mL (range 34.52–201.68), and the media 8-oxo-dG level was 180.44 pg/mL (range 57.50–676.20) in the first postnatal week. Seventeen cases had normal cUS and MRI results. The specific information is shown in Table 1.

**Table 1:** Clinical characteristics of cases with brain injury

Variable, *n* (%) or median (IQR)	Brain injury group, *N* = 58	No brain injury group, *N* = 17	*p*-Value
Gestational age (weeks)	30.38 ± 1.15	30.37 ± 1.39	0.979
Birth weight (g)	1499.69 ± 220.45	1560.88 ± 238.37	0.326
Male (%)	30 (51.7)	12 (70.6)	0.266
Premature rupture of membranes (%)	12 (20.7)	2 (11.8)	0.502
Abnormalbility of fetal attachment structure (%)	9 (15.5)	6 (35.3)	0.073
Mother’ age >30 years (%)	46 (79.3)	11 (64.7)	0.215
Cesarean section (%)	25 (43.1)	6 (35.3)	0.565
Asphxia (Apgar at 1, 5 and 10 min <7) (%)	17 (29.3)	5 (29.4)	1.000
Neonatal respiratory distress syndrome (%)	29 (50.0)	9 (52.9)	0.831
Mechanical ventilation (%)	17 (29.3)	6 (35.3)	0.638
Patent ductus arteriosus (%)	18 (31.0)	5 (29.4)	1.000
Necrotizing enterocolitis (%)	9 (15.5)	0 (0)	0.109
Infection (%)	13 (22.4)	5 (29.4)	0.536
Retinopathy of premature (%)	28 (48.3)	11 (64.7)	0.233

### 3.2 Plasma MBP and 8-oxo-dG levels

Ten patients with WMI had higher MBP levels compared to patients without WMI. However, there were no statistically significant differences in the PIVH group (Table 2).

**Table 2:** Plasma MBP and 8-oxo-dG levels

Parameter	*N*	MBP		8-oxo-dG (lg10)	
		Mean ± SEM	*p*-Value	Mean ± SEM	*p*-Value
**Brain injury**					
No	17	69.10 ± 12.00	0.364	2.13 ± 0.17	0.234
Yes	58	73.80 ± 20.12		2.21 ± 0.24	
**PIVH**					
No	19	73.11 ± 19.58	0.986	2.16 ± 0.18	**0.021**
I–II	46	72.77 ± 20.14		2.16 ± 0.24	
III–IV	10	71.87 ± 7.44		2.38 ± 0.16	
**WMI**					
No	65	69.45 ± 13.51	**0.034**	2.17 ± 0.22	**0.022**
Yes	10	94.09 ± 31.13		2.35 ± 0.22	

Sever PIVH was significantly associated with fewer infants with a high 8-oxo-dG level. The infants with mild hemorrhage and without PIVH had similar 8-oxo-dG levels. WMI was also significantly associated with high 8-oxo-dG levels (Table 2).

### 3.3 The relation between MBP and 8-oxo-dG and their prediction in WMI

**Figure 1:**
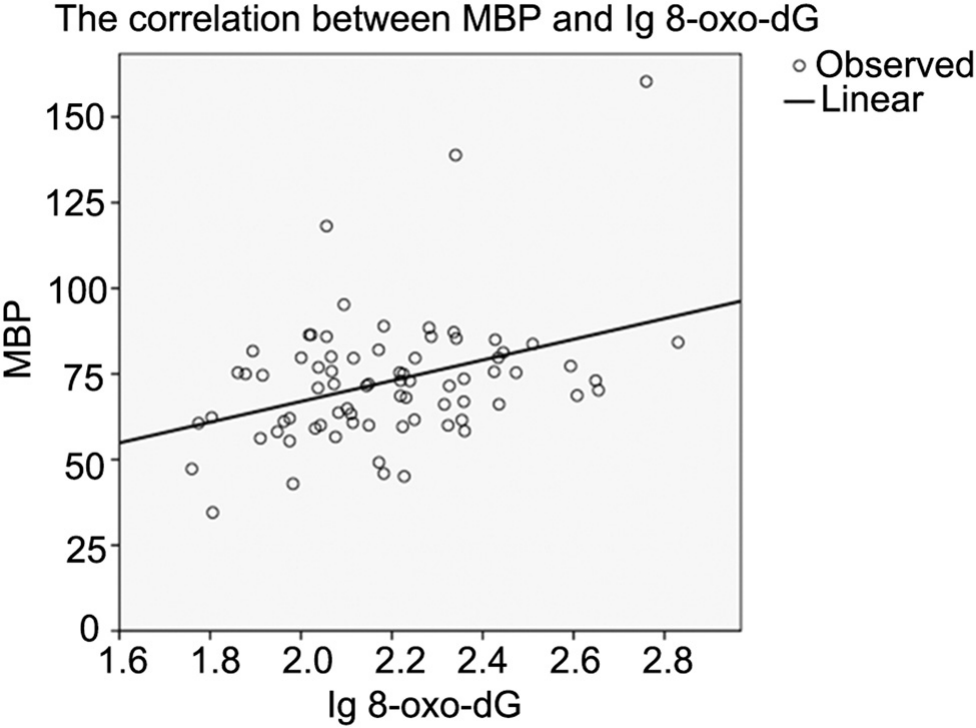
The correlation between MBP and Ig 8-oxo-dG.

Both the plasma levels of MBP and 8-oxo-dG in the no WMI and PIVH group were not correlated with gestational age. There was significant correlation between MBP and 8-oxo-dG (*r* = 0.390, *p* = 0.001). The linear regression equation: MBP value = 30.25lg8-oxo-dG + 6.49. The relation between MBP and 8-oxo-dG is shown in Figure 1.

**Figure 2:**
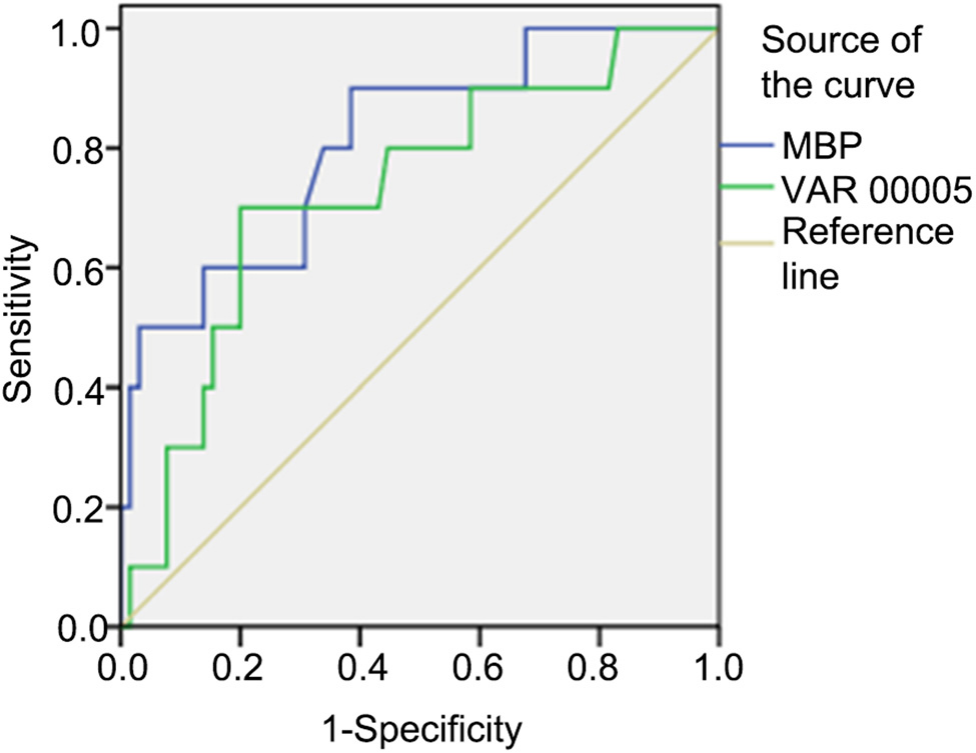
ROC curve analysis of MBP and 8-oxo-dG in WMI.

Due to the above significant relation of MBP and 8oxo-dG with WMI, an ROC curve analysis was performed to estimate the diagnostic potential of the neuro-biomarkers in WMI (Table 2 and Figure 2). We did not show the area under the curve (AUC) in the combination of the two biomarkers as AUC in MBP almost covered that in 8-oxo-dG. AUC was 0.811 (0.667–0.955) (*p* = 0.002) in MBP and 0.729 (0.562–0.897) (*p* = 0.002) in 8-oxo-dG. For MBP, when the cut-off point was 73.32 pg/mL, the sensitivity was 90% and the specificity was 61.54%, and PPV and NPV were 26.47 and 97.56%, respectively. For 8-oxo-dG, when the cut-off point was 211.45 pg/mL, the sensitivity was 70% and the specificity was 78.46%, and PPV and NPV were 33.33 and 94.44%, respectively. All data are shown in Table 3.

**Table 3:** The relation between MBP and 8-oxo-dG and their prediction in WMI

Indexes	MBP	8-oxo-dG
AUC (95% CI)	0.811 (0.667–0.955)	0.729 (0.562–0.897)
*p*-Value	0.002	0.020
Cut-off point (pg/mL)	73.32	211.45
Sensitivity	90	70
Specificity	61.54	78.46
NPV	97.56	94.44

## 4 Discussion

Preterm labor in the last trimester may disturb the rapid brain development. Preterm-related complications and associated medical care particularly interrupt the normal maturation and brain development in the first postnatal month [18]. Preterm infants face a significant risk of brain injury (e.g., intraventricular or germinal matrix hemorrhage and WMI) in the perinatal period, as well as potential long-term neurodevelopmental disabilities [19,20]. However, preterm children with brain injury lack specific clinical manifestations in the early days. Therefore, timely and accurate diagnosis of brain injury is of vital importance.

Currently, the diagnosis of brain injury in preterm infants commonly depends on neuroimaging, which is mainly performed by cUS and MRI. cUS should be the first choice of imaging modality for screening preterm children with brain injury since it provides a rapid and straightforward assessment of infants’ brains. Also, cUS is sensitive for the germinal matrix, intraventricular hemorrhage, hydrocephalus, as well as the focal cystic periventricular leukomalacia (PVL) in the preterm infant, while some subtle lesions and diffused PVL are indiscernible [21], but MRI and particularly diffusion-weighted image have a higher sensitivity for accurate diagnosis of WMI [22]. Nonetheless, unstable preterm infants need to be moved and endure noise for operation. Routine MRI can be done only in stable preterm infants or those at a corrected gestational age of 40 weeks, which may result in delayed intervention for brain injury. Consequently, it is essential to identify sensitive biomarkers for the early diagnosis of brain injury.

The most common preterm brain injuries in very and extreme preterm infants born in developed countries are periventricular–intraventricular hemorrhages (PIVH) and diffuse WMI. Due to the individual limitations related to cUS and MRI, these two examination methods could be combined to provide a more accurate diagnosis of brain injury. In this study, we used cranial B-ultrasound combined with MRI for the diagnosis and excluded the cases without cUS and MRI. Ten cases showed varying degrees of WMI, including periventricular hyperechoic, the diffuse abnormal signal in white matter area, and focal malacia. There were 56 cases with PIVH, including 13 cases with III–IV degrees.

There are two critical triggers of the brain injury, obstruction of myelin synthesis in developing brain and oxidative stress in preterm brain injury [11,15]. MBP that is essential for myelination and 8-oxo-dG that is relevant for oxidative stress has gained a lot of attention among research community as the specific markers of preterm brain injury. However, there is no normal range for the two neuro-markers at present. Since both MBP and 8-oxo-dG are important in the process of early brain development, our study assessed the role of these two biomarkers in the early postnatal age on the neurological outcomes in very preterm infants. We found that the most common brain abnormalities were WMI and PIVH. Plasma MBP levels markedly increased in WMI, while 8-oxo-dG levels were significantly elevated both in WMI and PIVH. Therefore, our study further verified that oligodendrocyte maldevelopment was strongly related to WMI in premature infants, while oxidative stress was closely related to both WMI and PIVH.

MBP is a major component of the central nervous myelin membrane. Pathological consults, such as hypoxia–ischemia and inflammation, may damage neuroglial cells followed by increased MBP levels in the bloodstream [8]. Chen et al. [16] reported that the serum MBP levels were significantly higher in the brain injury group. Also, Zhou et al. [23] demonstrated that the MBP serum levels in preterm infants were less than GA 34 weeks with PVL that was significantly increased in the first postnatal week. However, MBP levels in these infants with PIVH did not change substantially. In this study, the MBP levels in serum were significantly higher in the WMI group but not in the PIVH group, which was consistent with Huang’s previous report. These studies suggested that high MBP levels were related to poor myelination of oligodendrocyte, which might be the early risk factor of WMI.

8-oxo-dG is commonly used as a marker of oxidative stress-derived DNA damage. It reflects DNA repair enzyme activity and local antioxidant capacity. The level of 8-oxo-dG may reflect the severity of oxidative stress. In adult patients with neurodegenerative diseases, such as Alzheimer’s disease and Parkinson’s disease, 8-oxo-dG excretion is increased in urinary and CSF [24]. In children patients with various forms of central nervous system disorders, such as CNS infections, status epilepticus, and hypoxic–ischemic encephalopathy, 8-oxo-dG levels in CSF were found to evaluate significantly [25]. Several studies on newborns also verified that 8-oxo-dG is a useful marker for oxidative DNA damage. In this study, plasma serum 8-oxo-dG levels were both significantly higher in PIVH and WMI. These results provided further verification that preterm-related brain injury was closely related to oxidative DNA damage.

Previous studies have reported that oligodendroglial precursor (pre-OL) injury is the main cause of WMI [11]. Myelination process is the key step in pre-OL maldevelopment. Also, accumulation of reactive oxygen species and lack of antioxidative ability in preterm are considered important in the etiology of WMI [26,27]. Studies on both human and animal brain models verified that free radical injury contributes to the oligodendroglia dysfunction [28,29,30]. In this study, a significant correlation that was found between MBP and 8-oxo-dG indicated the existence of a close relationship between oxidative stress and maldevelopment of oligodendrocyte. This further verified that oxidative stress is implicated in the differentiation, myelination, and maturation of oligodendrocytes. The greater severity of oxidative stress is linked to the worse myelination. Myelination process in the brain is the morphological development that is directly related to brain functionality. Our results revealed a higher diagnostic efficiency of MBP compared with 8-oxo-dG in WMI. The negative predictive diagnosis of MBP and 8-oxo-dG was both high to 97.56 and 94.44%, respectively. This suggests that high levels of these two biomarkers in the brain indicate the tendency of brain injury in preterm infants.

This study assessed the early diagnostic efficiency of MBP and 8-oxo-dG in WMI and PIVH. The level of two biomarkers was both closely associated with WMI and PIVH in the very preterm infants. Yet this study has a few limitations: (1) the two sensitive markers were tested in the first postnatal week, while maybe some babies have got WMI and PIVH; (2) small sample size (low number of brain injury cases were included); and (3) due to the high drop-out rate during follow-up, the long-term neurological outcomes could not be assessed. Further studies should address the specific role of these neuro-markers in the preterm brain injury.

## 5 Conclusions

In the early postnatal period, high MBP levels indicated a possibility of WMI in the premature brain, while high 8-oxo-dG levels are closely related to both WMI and PIVH. Therefore, MBP and 8-oxo-dG may be potential neuro-markers of preterm brain injury.

## List of abbreviations

8-oxo-Dg: 8-oxo-deoxyguanosine
AUC: area under the curve
BW: birth weight
CNS: central nerve system
CSF: cerebrospinal fluid
cUS: cranial ultrasound
ELISA: enzyme-linked immunosorbent assay
GA: gestational age by weeks calculated from the last menstrual period and confirmed by physical examination
MBP: myelin basic protein
MRI: magnetic resonance imaging
NICU: neonatal intensive care unit
PIVH: periventricular–intraventricular hemorrhages
pre-OL: oligodendroglial precursor
PVL: periventricular leukomalacia
ROC: receiver–operator characteristic
WMI: white matter injury
